# Contributions of childhood adversities to chronic pain among mid-life employees

**DOI:** 10.1177/1403494820981509

**Published:** 2021-01-18

**Authors:** Aino Salonsalmi, Olli Pietiläinen, Eero Lahelma, Ossi Rahkonen, Tea Lallukka

**Affiliations:** Department of Public Health, University of Helsinki, Finland

**Keywords:** Adverse childhood experiences, chronic pain

## Abstract

*Aims:* Chronic pain is a notable burden on public health, with past and present factors contributing to it. This study aimed to examine the associations between childhood adversities and chronic pain. *Methods:* Data on seven childhood adversities, chronic pain and disabling pain were derived from questionnaire surveys conducted in 2000, 2001 and 2002 among 40- to 60-year-old employees (response rate of 67%) of the City of Helsinki, Finland. The study included 8140 employees (80% women). Logistic regression was used in the analyses, and the results are presented as odds ratios (OR) and their 95% confidence intervals (CI). Age, sex, the father’s education, the participant’s education, marital status, working conditions, sleep problems and common mental disorders were included as covariates. *Results:* In the age-adjusted models, childhood economic difficulties (OR=1.60, 95% CI 1.41–1.81), childhood illness (OR=1.74, 95% CI 1.45–2.08), parental divorce (OR=1.26, 95% CI 1.07–1.48), parental alcohol problems (OR=1.34, 95% CI 1.18–1.52) and bullying at school or among peers (OR=1.59, 95% CI 1.37–1.89) were associated with chronic pain. Working conditions, sleep problems and common mental disorders each slightly attenuated the associations between childhood adversities and chronic pain. Childhood economic difficulties among women (OR=1.72, 95% CI 1.40–2.10), childhood illness (OR=1.40, 95% CI 1.07–1.82) and bullying at school or by peers (OR=1.91 95% CI 1.48–2.46) were also associated with disabling pain. *Conclusions:* **Childhood adversities were associated with chronic pain in mid-life, and the associations mainly remained after adjustments. Investing in the well-being of children might prevent pain and promote well-being in mid-life.**

## Introduction

Chronic pain is a major burden on public health worldwide. Pain can significantly limit daily activities of life and work when it is disabling. Chronic pain is common among employees and in work life. It is associated with problems such as reduced work capacity [1] and sickness absence [2]. There is increasing evidence that childhood adversities contribute to various adult health outcomes [3,4]. When it comes to chronic pain, there is rather consistent evidence that childhood abuse and neglect are associated with later chronic pain [5], but studies on other childhood adversities are scarce, with somewhat inconclusive results.

Canadian studies found that an accumulation of childhood adversities [6,7], hospitalisation as a child and parental unemployment increased the risk of chronic back pain, whereas such adversities as being sent away from home and parental divorce did not [7]. A British study on a large birth cohort found that only part of the studied childhood adversities, such as maternal death and childhood economic difficulties, increased the risk of chronic pain [8]. Another British study reported that of the studied seven childhood adversities, only hospitalisation in childhood was associated with chronic widespread pain [9]. A Japanese study found that family violence and substance use were associated with chronic neck and back pain, whereas parental death and divorce and parental mental disorder showed no associations [10]. A US study reported that maternal depression was associated with chronic pain [11].

There are various potential mechanisms for how childhood adversities might contribute to adult chronic pain. First, childhood illness might continue in adulthood or be associated with adult health problems causing chronic pain. Second, childhood chronic stress can modify the development of the nervous, endocrine and immune systems, resulting in altered cognitive, social and emotional functioning and chronic physiological damage [12]. Thus, childhood adversities might be associated with adult diseases that cause or are associated with chronic pain or modify pain via influencing factors such as adult socio-economic status [13], marital status [14], health behaviours [15] and sleep problems [16] that are, in turn, determinants of chronic pain. Childhood adversities can be seen as distal determinants of pain that work together with more proximal determinants of pain (e.g. factors that are temporally closer to pain).

The aim of this study was to examine whether childhood adversities are associated with chronic pain among mid-life employees. Another aim was to examine whether the father’s education, the participant’s education, marital status, sleep problems and common mental disorders contribute to the associations. As chronic pain is common among employees, a further aim was to examine if childhood adversities were also associated with disabling pain, which is an even more relevant contributor to work ability.

## Methods

This study is part of the Helsinki Health Study on employees of the City of Helsinki in Finland [17]. The City of Helsinki is the largest employer in Finland, with about 37,000 employees in diverse fields, including teachers, lawyers, nurses, doctors, garden workers and bus drivers. The majority of the employees (76%) are women, which corresponds to the Finnish municipal sector.

The baseline survey was conducted in 2000, 2001 and 2002 via mailed questionnaires to employees of the City of Helsinki who reached the age of 40, 45, 50, 55 or 60 during those years. In all, 8960 employees out of a target population of 13,346 participated, yielding a response rate of 67%. After exclusion due to missing information on chronic pain (*n*=303) and covariates (*n*=583), the present study includes 8140 employees, of whom 1638 were men and 6502 were women. In addition, item non-response varied between 808 and 953 answers concerning variables on childhood adversities, and thus the final analyses included somewhat fewer participants. The item non-response on childhood adversities partially overlapped with other non-response, and the final numbers of participants in the different analyses are presented in Table I. The analysis regarding disabling pain included 3769 participants.

**Table I.** Distribution of chronic pain and childhood adversities and prevalence of chronic pain in mid-life by childhood adversities.

	*n*	%	Prevalence of chronic pain (%)
Sex			
Women	6502	80	30
Men	1638	20	24
Childhood economic difficulties			
No	6093	81	26
Yes	1394	19	37
Childhood illness			
No	6840	92	27
Yes	564	8	39
Parental divorce			
No	6563	87	27
Yes	837	11	30
Parental death			
No	6413	86	28
Yes	1020	14	30
Parental mental illness			
No	6903	94	28
Yes	408	6	32
Parental alcohol problems			
No	6007	80	27
Yes	1489	20	32
Bullying at school or by peers			
No	6743	91	27
Yes	636	9	35

The non-response analyses of the Helsinki Health Study found that responses at baseline tended to be lower among younger employees, those with lower occupational positions and those with longer sickness absence during the study year, although the differences were minor and not fully consistent [17].

The Ethics Committee of the Department of Public Health at the University of Helsinki and the Ethics Committee of the health authorities at the City of Helsinki approved the study.

### Childhood adversities

Data on childhood adversities before the age of 16 were derived from the baseline questionnaire survey, and each question allowed two response alternatives (yes/no). The adversities were serious or long-term illness during childhood, parental divorce, parental death, the father or mother having mental health problems, father or mother consuming alcohol to the degree that it caused problems at home, significant economic difficulties in the family and having been bullied at school or by peers. Similar questions on childhood adversities have been used in previous studies [18].

### Pain measures

Data on chronic pain were derived from the baseline survey. There was a question about whether the respondent had aches or pain at the moment. A further question inquired about when the pain started, with response alternatives of ‘not more than three months ago’ and ‘more than three months ago’. Those with pain that started more than three months prior were classified as having chronic pain, and others formed the reference group. The definition of chronic pain followed the recommendation of the International Association for the Study of Pain [19].

For those participants reporting pain (*n*=3769), further questions on disabling pain were addressed, and an additional analysis of the associations between childhood adversities and disabling pain was performed. Disabling pain was measured by a disability subscale of von Korff’s Chronic Pain Grade questionnaire [20]. The questionnaire measures disability related to daily activities, work ability and leisure activities, and enquires about disability days during the past six months. The cut-off point for disabling pain was set between disability points 2 and 3.

### Covariates

**Figure 1.**
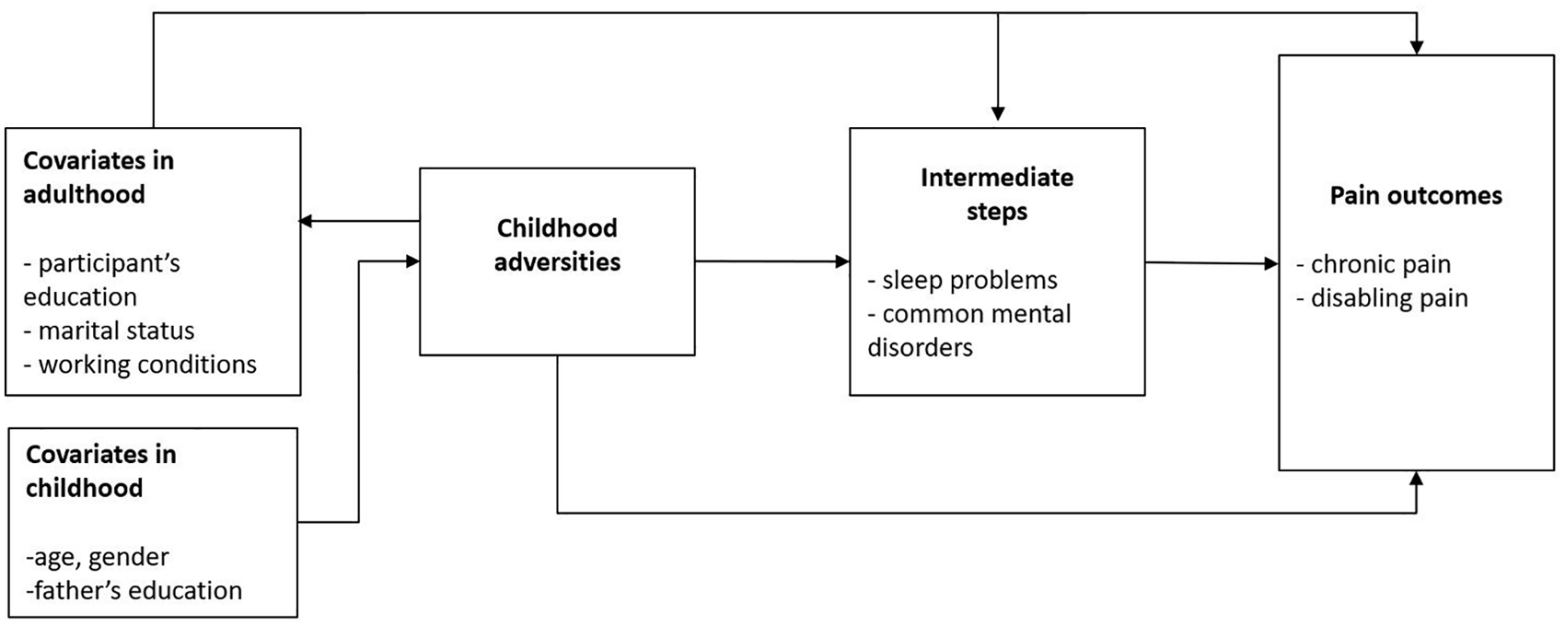
Directed acyclic graph of the assumed associations and pathways between childhood adversities, chronic pain and covariates. Childhood adversities were inquired retrospectively at the same time as pain outcomes and covariates.

Sex and age at the time of the survey were included as covariates. The father’s education was inquired by a question with six response alternatives and divided into three classes: ‘low’ (elementary school or part of it or intermediate school), ‘mid-level’ (vocational school or matriculation examination or college-level training) and ‘high’ (polytechnic or university degree). Marital status was divided into three groups: single, married or cohabiting and widowed or divorced. The participant’s education was divided into three groups: elementary school or intermediate school, vocational school, matriculation examination or college-level training and polytechnic or university degree. Working conditions consisted of self-reported physical and mental strenuousness of the work, each measured by a single-item question with four response alternatives, ranging from ‘very light work’ to ‘very strenuous work’. Sleep problems were measured by the four-item Jenkins Sleep Questionnaire [21]. The items included three subdomains of sleep problems, namely difficulties initiating and maintaining sleep and nonrestorative sleep. Those reporting any of the inquired sleep disturbances in 15–28 nights during the previous four weeks were defined as having sleep problems. Common mental disorders were measured by the 12-item General Health Questionnaire [22] covering, for example, symptoms of anxiety and depression. Those scoring three or more were classified as having common mental disorders. Assumed associations and pathways between childhood adversities, chronic pain and covariates are shown in Figure 1.

### Statistical methods

The associations between childhood adversities and chronic pain were examined by logistic regression analysis. Those with no childhood adversities served as reference groups. Chronic pain and disabling pain served as the dependent variables, and childhood adversities served as independent variables. Interactions for sex were tested (*p*<0.1), and the only significant interaction (*p*=0.033) concerned childhood economic difficulties and disabling pain. Thus, this analysis was performed separately for men and women. Otherwise, sexes were pooled. First, we fitted models adjusted for age and sex. Then, the father’s education, the participant’s education and marital status, working conditions, sleep problems and common mental disorders were added to the models one at a time. The results are presented as odds ratios (OR) and their 95% confidence intervals (CI). SAS v9.4 (SAS Institute, Cary, NC) was used to perform the analyses.

## Results

Chronic pain was reported by 24% of men and 30% of women. The prevalence of childhood adversities varied between 6% (parental mental illness) and 20% (parental alcohol problems; Table I). Parental alcohol problems and childhood economic difficulties were the most common childhood adversities. The prevalence of chronic pain varied according to childhood adversities, with those reporting childhood adversities having more chronic pain.

Childhood economic difficulties were associated with chronic pain in mid-life (OR=1.60, 95% CI 1.41–1.81; Table II). Adjusting for the father’s education or for the participant’s education and marital status had no effect, whereas adjusting for working conditions, sleep problems and common mental disorders each slightly attenuated the association. Childhood illness was associated with chronic pain in mid-life (OR=1.74, 95% CI 1.45–2.08). The father’s education, the participant’s education, marital status and working conditions had no effect on the association, whereas sleep problems and common mental disorders somewhat attenuated it.

**Table II.** Associations between childhood adversities and chronic pain in mid-life.

	Model 1=Age and sex	Model 1+father’s education	Model 1+the participant’s education+marital status	Model 1+working conditions	Model 1+sleep problems	Model 1+common mental disorders
Childhood economic difficulties	1.60 (1.41–1.81)	1.56 (1.38–1.77)	1.55 (1.37–1.75)	1.49 (1.31–1.69)	1.47 (1.29–1.67)	1.48 (1.30–1.68)
Childhood illness	1.74 (1.45–2.08)	1.74 (1.45–2.08)	1.77 (1.47–2.11)	1.68 (1.40–2.02)	1.56 (1.30–1.88)	1.59 (1.33–1.92)
Parental divorce	1.26 (1.07–1.48)	1.26 (1.07–1.48)	1.22 (1.03–1.43)	1.26 (1.07–1.49)	1.21 (1.02–1.42)	1.26 (1.07–1.49)
Parental death	1.02 (0.88–1.18)	1.00 (0.87–1.16)	0.98 (0.85–1.14)	1.00 (0.86–1.16)	1.01 (0.87–1.17)	1.01 (0.87–1.18)
Parental mental illness	1.24 (1.00–1.55)	1.25 (1.01–1.56)	1.26 (1.02–1.57)	1.16 (0.93–1.45)	1.11 (0.89–1.39)	1.11 (0.89–1.39)
Parental alcohol problems	1.34 (1.18–1.52)	1.32 (1.16–1.50)	1.31 (1.15–1.48)	1.28 (1.13–1.56)	1.27 (1.11–1.44)	1.27 (1.12–1.44)
Bullying at school or by peers	1.59 (1.37–1.89)	1.57 (1.32–1.87)	1.56 (1.31–1.86)	1.49 (1.25–1.78)	1.44 (1.20–1.72)	1.46 (1.22–1.74)

Data shown are odds ratios and their 95% confidence intervals.

Parental divorce was weakly associated with chronic pain (OR=1.26, 95% CI 1.07–1.48). None of the covariates contributed to the association. Parental death was not associated with chronic pain in mid-life.

Parental mental illness was weakly associated with chronic pain in models adjusted for the father’s education and the participant’s education and marital status.

Parental alcohol problems were associated with chronic pain (OR=1.34, 95% CI 1.18–1.52), and the association remained after all of the different adjustments. Having been bullied at school or by peers was also associated with chronic pain (OR=1.59, 95% CI 1.37–1.89). Working conditions, sleep problems and common mental disorders each somewhat attenuated the association.

Of those with pain, 21% had disabling pain (Table III). Childhood economic difficulties among women (OR=1.72, 95% CI 1.40–2.10), childhood illness (OR=1.40, 95% CI 1.07–1.82) and having been bullied at school or by peers (OR=1.91, 95% CI 1.48–2.46) were associated with disabling pain (Table III).

**Table III.** Distribution of chronic widespread pain and the associations between childhood adversities and disabling pain in mid-life.

**Distribution of disabling pain among those with pain**		
	***n***	**%**
No	2983	79
Yes	786	21
	**Age and sex**	
Childhood economic difficulties^a^		
Women	1.72 (1.40–2.10)	
Men	0.97 (0.58–1.62)	
Childhood illness	1.40 (1.07–1.82)	
Parental divorce	1.18 (0.91–1.53)	
Parental death	1.14 (0.90–1.44)	
Parental mental illness	0.97 (0.69–1.38)	
Parental alcohol problems	1.19 (0.97–1.45)	
Bullying at school or by peers	1.91 (1.48–2.46)	

Data shown are odds ratios and their 95% confidence intervals.

a Women and men analysed separately because of a significant interaction for sex.

## Discussion and conclusions

Of the seven studied childhood adversities, five –childhood economic difficulties, childhood illness, parental divorce, parental alcohol problems and bullying at school or among peers – were associated with chronic pain. Childhood economic difficulties, childhood illness and bullying at school or by peers showed the strongest associations, and the same childhood adversities were associated with disabling pain. Adjusting for the father’s education and for marital status and the participant’s education had virtually no contributions to the associations. Working conditions, sleep problems and common mental disorders somewhat attenuated the associations, which mainly remained after the adjustments.

Childhood illness showed the strongest association with chronic pain in mid-life. Previous studies have examined hospitalisation in childhood, portraying a rather severe period of illness, and found it to be associated with chronic widespread pain [9] and back pain [7] in adulthood. One explanation for this association might be that there are pathways from childhood illness to poor health as an adult. In the present study, however, the participants were mid-life employees, and those with the most disabling illnesses might drop out of the workforce before mid-life. None of the covariates was able to abolish the association, but sleep problems and common mental disorders slightly attenuated it.

Childhood economic difficulties were one of the childhood adversities showing the strongest associations with chronic pain. Adjusting for either the father’s or the participant’s education did not abolish the association, and our study was thus in line with a British study that showed that economic difficulties were associated with pain after adjusting for both childhood and adult socio-economic status [8]. Also, another Finnish study focusing on back pain reported that childhood socio-economic circumstances were associated with adult pain, independent of current socio-economic status [23]. Of the other studied covariates, working conditions, sleep problems and common mental disorders slightly attenuated the association. A shortage of material resources might have influenced non-material resources and family environments that, in turn, shaped adult responses to pain and its more proximal determinants.

The third childhood adversity that showed a rather strong association with chronic pain was bullying at school or by peers. Adjustments for covariates had only small contributions to the association. Having been bullied by peers has been shown to be associated with adult mental health problems [24], but its contributions to other health outcomes remain unclear. A German study examined pain outcomes with a short duration of pain and found that having been bullied by peers was associated with overall pain, bodily pain, headaches and back or neck pain [25].

Parental alcohol problems and parental mental illness have both been associated with offspring mental health problems [26,27], but studies on their association with adult chronic pain are scarce. A US study found that maternal depression was associated with chronic pain [11], whereas a Japanese study reported that parental mental illness was not associated with pain, but parental substance disorder was [10]. British and Canadian studies found no association between parental alcohol problems and pain [7,8]. In the present study, parental alcohol problems were associated with chronic pain, whereas the association between parental mental illness and chronic pain was weak.

Parental death was not associated with chronic pain, and the association between parental divorce and chronic pain was weak. Findings from previous studies have been somewhat inconclusive. In a Japanese study, neither parental divorce nor death was associated with pain [10]. In a Canadian study, parental divorce was not associated with back pain [7], and in a British study, loss of a parent was not associated with chronic widespread pain [9]. In another British study, death of the mother was associated with chronic pain, but death of the father was not [8]. The heterogeneity of the results suggests that parental death or divorce might not be directly related to pain, but they might contribute to other more proximal determinants of chronic pain. In addition, other childhood adversities might confound the associations. In the present study, adjusting for none of the covariates contributed to the association.

Chronic pain was rather common among the studied employees, but the measure did not separate whether the employee was able to carry out her/his daily activities. Thus, we further examined the association of childhood adversities and disabling pain. Of the childhood adversities, childhood economic difficulties among women, childhood illness and having been bullied were associated with disabling pain. Thus, the results supported the findings from the analyses concerning chronic pain, as childhood adversities that showed the strongest associations with chronic pain were also associated with disabling pain.

Despite previous studies, the mechanisms behind the association of childhood adversities and adult chronic pain remain largely unclear. The interest has often been in the idea that childhood adversities manifest as pain, which has been seen as a mental health problem by origin. The mechanism of pain becoming chronic is a complex and not yet fully understood process affected by diverse biological, psychosocial and social factors such as severity of acute pain, sleep problems and catastrophising thoughts about pain such as ‘the pain will never end’ or ‘the pain may get worse’. It has been suggested that childhood adversities influence adult health by having direct, long-lasting effects (latency model), or they may have influence through adult conditions (pathway model) or by an accumulation of factors acting over a lifetime (cumulative model) [28].

In our study, several covariates were included to shed light on the studied associations. The father’s education itself was associated with chronic pain (data not shown) but did not contribute to the associations. The participant’s education and marital status had virtually no contributions. Socio-economic status and marital status were thus unlikely to transmit the associations between childhood adversities and chronic pain. Working conditions, sleep problems and common mental disorders slightly attenuated all of the associations, with the exception of the rather weak association between parental divorce and chronic pain. The contribution of common mental disorders was not substantial and did not support the idea of chronic pain originating from mental health reasons. Analyses were also run adjusting for all studied covariates simultaneously, and the associations between childhood adversities and chronic pain were attenuated but remained (data not shown). As the simultaneous adjustment included a large set of variables, it is difficult to interpret the effects, and there is a risk of over-adjustment. All in all, together with the previous evidence, the results suggest potential latent routes from childhood adversities to chronic pain. In addition, childhood adversities acting together with proximal determinants of chronic pain such as adult working conditions, sleep problems and common mental disorders gained some evidence. It might be that childhood adversities predispose to sleep problems and mental health problems that, in turn, contribute to pain becoming chronic. The contribution of different childhood adversities to chronic pain might be transmitted via different routes. Regarding part of the childhood adversities, especially parental alcohol drinking and parental mental health problems, the association might be due to offspring predisposition rather than childhood adversity, as genetic heritability plays a role.

Previous studies have suggested that multiple childhood adversities might have a multiplicative contribution to adult health outcomes [3]. Thus, the association between the sum score of childhood adversities and adult chronic pain was examined in additional analyses presented in Supplemental Table S1. The associations were stronger for multiple adversities (OR=1.85, 95% CI 1.34–2.55 for those with four or more adversities; OR=1.65, 95% CI 1.45–1.88 for those with two to three adversities; and OR=1.24 95%, CI 1.11–1.40 for those with one adversity only). Also, the associations here remained after all adjustments, although sleep problems and mental disorders slightly attenuated the associations. Only 2% of the participants reported four or more adversities, whereas 18% had two to three childhood adversities.

The advantages of this study include a large data set and the possibility to control for various covariates. The main limitation of the study was its cross-sectional design with retrospective reports of childhood adversities. A review on the validity of adult reports on childhood adversities reported that there is a substantial rate of false-negative reports, whereas false-positive results are probably rare [29]. Non-recurring events such as parental death or divorce might be correctly reported more often than childhood adversities requiring subjective interpretation such as bullying at school or by peers. Under-response among younger employees, among those with lower occupational positions and among those with longer sickness absence might have biased the results, as age, low occupational position and sickness absence all associate with chronic pain. In addition, it might be that those with childhood adversities were selected as non-respondents due to the intimate character of the questions. People with the severest childhood adversities may have dropped out of the workforce, which might dilute the findings.

In conclusion, this study showed that childhood adversities were associated with chronic pain in mid-life. Neither the father’s nor the participant’s education contributed to the association, whereas working conditions, sleep problems and common mental disorders had small contributions. The association between retrospective childhood adversities and current chronic pain is temporally distant and complex, but the results suggest that adversities in the early life course play a role even in mid-life. In light of our study, investing in the well-being of children and their families is likely important and might promote well-being and lower the risk of chronic pain in mid-life.

## Supplemental Material

sj-pdf-1-sjp-10.1177_1403494820981509 – Supplemental material for Contributions of childhood adversities to chronic pain among mid-life employees Click here for additional data file. Supplemental material, sj-pdf-1-sjp-10.1177_1403494820981509 for Contributions of childhood adversities to chronic pain among mid-life employees by Aino Salonsalmi, Olli Pietiläinen, Eero Lahelma, Ossi Rahkonen and Tea Lallukka in Scandinavian Journal of Public Health
