# Supplementary material for: Contributions of childhood adversities to chronic pain among mid-life employees
Source: Scand J Public Health. 2021 Jan 18;50(3):333–9. doi: 10.1177/1403494820981509 (PMC9096588; doi:10.1177/1403494820981509)
Supplement: sj-pdf-1-sjp-10.1177_1403494820981509 – Supplemental material for Contributions of childhood adversities to chronic pain among mid-life employees [file sj-pdf-1-sjp-10.1177_1403494820981509.pdf]

Table S1. Associations between childhood adversity sum score and chronic pain in midlife. Odds ratios and their 95% confidence intervals.

	Model 1 = Age and gender	Model 1 + father's education	Model 1 + the participant's education + marital status	Model 1 + working conditions	Model 1 + sleep problems	Model 1 + common mental disorders
No childhood adversity (n=4129)	1.00	1.00	1.00	1.00	1.00	1.00
1 childhood adversity (n=2249)	1.24 (1.11–1.40)	1.23 (1.09–1.38)	1.22 (1.09–1.37)	1.20 (1.07–1.35)	1.19 (1.05–1.34)	1.20 (1.06–1.35)
2–3 childhood adversities (n=1459)	1.65 (1.45–1.88)	1.61 (1.41–1.83)	1.60 (1.40–1.82)	1.55 (1.36–1.77)	1.51 (1.32–1.72)	1.54 (1.35–1.75)
4 or more childhood adversities (n=175)	1.85 (1.34–2.55)	1.84 (1.33–2.54)	1.76 (1.28–2.44)	1.72 (1.24–2.38)	1.55 (1.11–2.15)	1.61 (1.16–2.24)
